# COVID-19 Vaccination Is Safe among Mast Cell Disorder Patients, under Adequate Premedication

**DOI:** 10.3390/vaccines10050718

**Published:** 2022-05-04

**Authors:** Tiago Azenha Rama, Joana Miranda, Diana Silva, Luís Amaral, Eunice Castro, Alice Coimbra, André Moreira, José Luís Plácido

**Affiliations:** 1 Serviço de Imunoalergologia, Centro Hospitalar Universitário São João, 4200-319 Porto, Portugal; joana.raquel.miranda@chsj.min-saude.pt (J.M.); disolha@med.up.pt (D.S.); luis.miguel.amaral@chsj.min-saude.pt (L.A.); eunice.castro@chsj.min-saude.pt (E.C.); alice.coimbra@chsj.min-saude.pt (A.C.); andremoreira@med.up.pt (A.M.); jose.placido@chsj.min-saude.pt (J.L.P.); 2 Serviço de Imunologia Básica e Clínica, Departamento de Patologia, Faculdade de Medicina, Universidade do Porto, 4200-319 Porto, Portugal; 3 EPIUnit—Institute of Public Health, University of Porto, 4050-600 Porto, Portugal; 4 Laboratory for Integrative and Translational Research in Population Health (ITR), 4050-600 Porto, Portugal

**Keywords:** COVID-19 vaccines, mastocytosis, clonal mast cell activation syndrome, anaphylaxis

## Abstract

Reported cases of anaphylaxis following COVID-19 vaccination raised concerns about the safety of these vaccines, namely in patients suffering from clonal mast cell (MC) disorders—a heterogenous group of disorders in which patients may be prone to anaphylaxis caused by vaccination. This study aimed to assess the safety of COVID-19 vaccines in patients with clonal MC disorders. We performed an ambidirectional cohort study with 30 clonal MC disorder patients (n = 26 in the prospective arm and n = 4 in the retrospective arm), that were submitted to COVID-19 vaccination. Among these, 11 (37%) were males, and median age at vaccination date was 41 years (range: 5y to 76y). One patient had prior history of anaphylaxis following vaccination. Those in the prospective arm received a premedication protocol including H1- and H2-antihistamines and montelukast, while those in the retrospective arm did not premedicate. Overall, patients received a total of 81 doses, 73 under premedication and 8 without premedication. No MC activation symptoms were reported. COVID-19 vaccination seems to be safe in patients with clonal mast cell disorders, including those with prior anaphylaxis following vaccination. Robust premedication protocols may allow for vaccination in ambulatory settings.

## 1. Introduction

Vaccines against the severe acute respiratory syndrome coronavirus 2 (SARS-CoV-2) have shown to be very effective in the prevention of morbidity and mortality of acute COVID-19 [1,2]. Four vaccines are currently authorized for use in the European Union: mRNA vaccines BNT162b2 (Pfizer-BioNTech) and mRNA-1273 (Moderna), and viral vector vaccines ChAdOx1 nCoV-19 (Astra-Zeneca) and Ad26.COV2.S (Johnson & Johnson) [3]. While anaphylaxis was not observed during phase III trials for all of these vaccines, trials excluded participants with a history of allergic reaction to any component of the vaccines [4,5,6,7]. At the beginning of the mass vaccination campaign, two cases of anaphylaxis were reported in healthcare workers with prior history of anaphylaxis, in the United Kingdom [8]. These events raised concerns over the safety of these vaccines, which came under heavy scrutiny from that moment forth. Since then, several studies have shown these vaccines to be safe, with an incidence of anaphylaxis ranging from 7.91 [9] to 10.67 [10] cases per million doses; which is lower than that reported for rabies, tick-borne encephalitis, measles-mumps-rubella-varicella, and human papillomavirus vaccines [10]. mRNA vaccines seem to be overall safer regarding anaphylaxis than their viral vector counterparts [11].

Mechanisms for immediate hypersensitivity/anaphylaxis to these vaccines are still a matter of debate, and may include IgE hypersensitivity to excipients (notably, polyethylene glycol, PEG/polysorbate [9], and tromethamine [12]), complement anaphylatoxin-derived mast cell (MC) activation [13] or direct MC activation through membrane or intracytoplasmic pattern recognition receptors (Toll-like receptors 3, 7 and 8 [9], and retinoic-acid-inducible gene-1, RIG-1 [14]). One might intuitively link the latter two mechanisms to an increased risk of immediate hypersensitivity/anaphylaxis in patients with clonal MC disorders, a heterogeneous group of diseases characterized by overactivation (monoclonal MC activation syndromes, MMAS) [15] and accumulation of clonal MC in one or more tissues (mastocytosis) [16]. These patients show not only an increased risk for anaphylaxis [17], but also an increased risk of exacerbation/elicitation of MC activation caused by vaccination, especially in children with cutaneous mastocytosis (CM) [18,19,20,21]. The aforementioned anaphylactic reactions raised concern among patients and prompted several authors to publish case reports and series of clonal mast cell disorder patients that underwent COVID-19 vaccination [22,23,24,25,26] with conflicting results. Of these, three studies reported no MC activation-compatible symptoms [22,23,24] (cumulative n = 139 patients), one reported mild symptoms in 2/73 patients [26], and another study reported 10 reactions compatible with MC activation out of 130 patients [25]. None referred to previous reactions to vaccines, and all were based on the vaccination of adults.

Herein, we aimed to assess safety of COVID-19 vaccines in patients with clonal MC disorders, including children and a patient with prior anaphylaxis following vaccination.

## 2. Materials and Methods

### 2.1. Study Design

**Figure 1:**
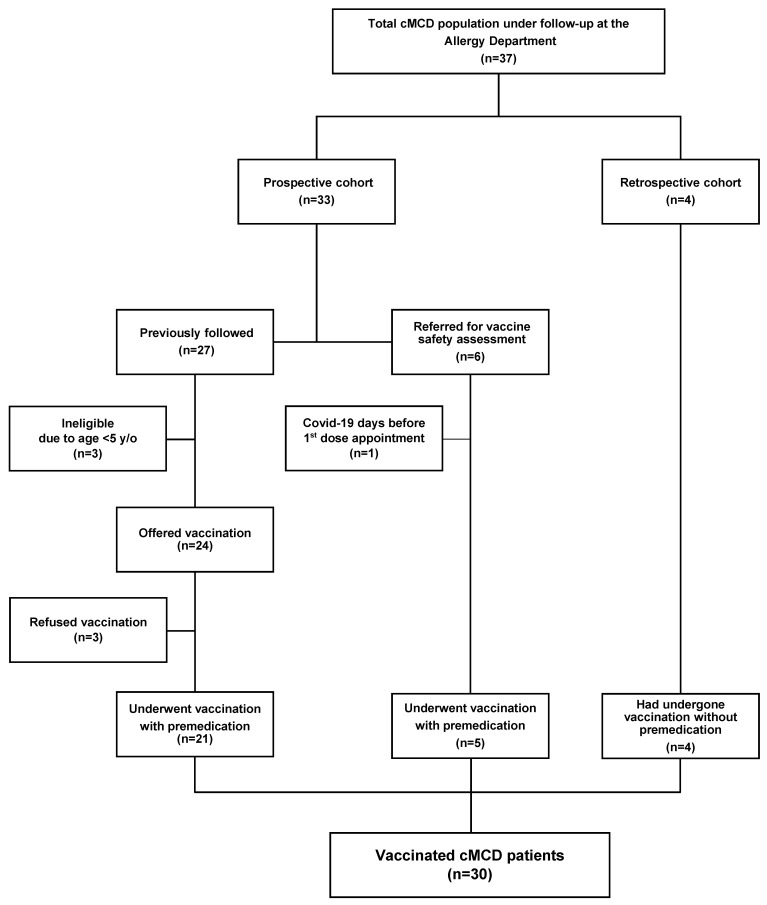
Flow chart of mastocytosis patients that underwent vaccination. Legend: cMCD, clonal mast cell disorders; y/o, years old.

We performed an ambidirectional cohort study with MMAS/mastocytosis patients (n = 36) diagnosed as per the WHO criteria [27] and followed at our University Hospital Center’s Allergy and Clinical Immunology department. The prospective arm of the study included those previously followed (n = 27) or referred to our department during the vaccination campaign for vaccine safety assessment (n = 6). The retrospective arm included those who were referred during the vaccination campaign and that had already been vaccinated (n = 4). For the prospective arm, inclusion criteria comprised eligibility for vaccination (i.e., age over 5 years old and absence of COVID-19 during the prior 3 months), while refusing COVID-19 vaccination was the only exclusion criterion. Among those previously followed, 4 did not comply with the inclusion criteria and 3 were excluded due to vaccination refusal (Figure 1). As such, 26 patients were included in the prospective and 4 in the retrospective arms of the study (Figure 1).

All patients provided consent to participate in the study.

### 2.2. Study Population 

A total of 30 patients underwent COVID-19 vaccination, of whom 11 (37%) were males. Median age at onset was 25 years (range: 2 months to 76 years), and 41 years (range: 5 years to 76 years) at the date of first vaccination (Table 1). 

Overall, 5 (17%) patients had cutaneous mastocytosis, 7 (23%) had mastocytosis in the skin, 12 (40%) had indolent SM, 2 (7%) had bone marrow mastocytosis, 1 (3%) had smoldering SM, 1 (3%) had SM with an associated hematologic neoplasm (myelodysplastic syndrome) and 1 (3%) had a monoclonal mast cell activation syndrome (MMAS). Concerning clinical manifestations, 24 (80%) had mastocytosis skin lesions, 23 (77%) had cutaneous manifestations (pruritus, flushing, lesion flareups or angioedema), 20 (67%) had gastrointestinal manifestations (heartburn, gastroesophageal reflux, abdominal pain, diarrhea), 14 (47%) had cardiovascular manifestations (orthostatic hypotension, recurrent tachycardia, presyncope/syncope) and 9 (30%) had anaphylaxis. Triggers for MC activation episodes included drugs in 9 (30%) (including anaphylaxis following prior vaccination with measles–mumps–rubella, that later tolerated other vaccines), Hymenoptera sting in 1 (3%), foods in 3 (10%) and idiopathic causes in 5 (17%) patients. 

### 2.3. Definitions and Diagnostic Procedures

Diagnosis of systemic mastocytosis/MMAS was based on the WHO diagnostic criteria, taking morphological, histopathological/immunohistochemical, immunophenotypic, molecular and analytical (serum baseline tryptase, ImmunoCAP Tryptase, Phadia/Thermo Fisher Scientific Inc., Uppsala, Sweden) data into account [15,16,27,28,29]. Regarding the molecular criteria, *KIT*^D816V^ mutation was assessed by allele-specific polymerase chain reaction (ASOqPCR), in peripheral blood, bone marrow or both. Children and adolescents with biopsy-proven mastocytosis skin lesions were diagnosed with cutaneous mastocytosis, as systematic performance of BM studies is not indicated at these ages. Adult patients with skin lesions in whom SM could not be confirmed/ruled out (incomplete or absent BM studies) were categorized as mastocytosis in the skin (MIS) [27]. Onset of mastocytosis was defined as the date of appearance of mastocytosis skin lesions, first episode of anaphylaxis or incidental detection of MC aggregates in BM study (in one patient) [30].

Blood tests performed at diagnosis and at follow-up included: complete blood cell count and differential, routine biochemistry, and both total and specific serum IgE levels (ImmunoCAP total IgE, Phadia/Thermo Fisher Scientific Inc.). Specific IgE levels were measured whenever adequate (ImmunoCAP allergen components, Phadia/Thermo Fisher Scientific Inc.). In addition, skin tests (e.g., skin prick or intradermal tests) were performed with specific triggers (e.g., Hymenoptera venom, aeroallergens, foods and drugs), whenever appropriate. Atopy was defined as positive specific IgE or a positive skin test for aeroallergens or foods (32). Specifically, among those with MC activation episodes due to drugs, only one had a history of potential excipient hypersensitivity (flushing and presyncope hours after taking high dose IV methylprednisolone acetate). This patient underwent skin testing (skin prick and intradermal tests) with methylprednisolone acetate, methylprednisolone succinate, PEG 1500 (ROXALL, Medizin GmbH, Hamburg, Germany), PEG 3350, dexamethasone, triamcinolone acetonide and polysorbate 20, all of which were negative. However, she was not submitted to an oral drug challenge with PEG.

### 2.4. Vaccination and Premedication Procedures

All patients in the prospective arm were prescribed a premedication protocol, which included H1 (bilastine 20 mg or ebastine 10 mg in those over 12 y/o and rupatadine 5 mg in the 5 y/o) and H2 (famotidine 40 mg in those over 12 y/o and 20 mg in the 5 y/o) antihistamines 1 h before, and montelukast (10 mg in those over 14 y/o and 5 mg in the 5 y/o) 24 and 1 h before vaccination, as we have previously reported [22]. Patients in the prospective arm were recommended for vaccination in a hospital that included an intensive care unit and were subsequently contacted by TAR to assess them for immediate or delayed-onset MC activation symptoms.

## 3. Results

A total of 81 doses were administered to 30 patients. In the prospective arm, 23/26 patients underwent vaccination in our hospital center, under a 30 min surveillance from allergists, for the first 2 doses. Among the remaining, 2/3 were vaccinated in ambulatory vaccination centers and 1/3 was vaccinated in another hospital. The third dose was administered to 16/26 patients, of which 7 (44%) were administered in ambulatory vaccination centers. A total of 12/29 patients only received 2 doses, 6 (50%) because they had COVID-19 and 6 (50%) awaiting recall for the third dose. The 5 year-old had COVID-19 shortly after receiving the first dose and had not received the second dose because of that. A total of 73 doses were administered: 24/26 received BNT162b2 30 µg (68 doses), 1/26 received 1 dose of BNT162b2 30 µg and 2 of mRNA-1273 100 µg, and the 5 year-old received BNT162b2 10µg. None of the 26 patients in this arm showed MC activation symptoms. All patients in the retrospective arm were vaccinated in ambulatory vaccination centers, all received the BNT162b2 (8 doses: 3 in 2 and 2 in 1 patient) without premedication, and all denied MC activation symptoms.

Overall, 11 patients had COVID-19, among whom 3 had not been vaccinated (2 were later vaccinated with 2 doses and 1 was not vaccinated), 1 had received 1 dose, 4 had received 2 doses, and 3 had received 3 doses. All patients had mild COVID-19, and only two displayed long-term manifestations (anosmia/hyposmia in both).

## 4. Discussion

EU-authorized COVID-19 vaccines exert their action through the production and later recognition by the immune system of the SARS-COV-2 envelope spike (S) glycoprotein, aiming for cellular and humoral (neutralizing) immune responses. Different platforms differ on the vehicle: in mRNA vaccines, S-encoding mRNA is vehiculated within a PEGylated nanolipid envelope, which prevents mRNA degradation and facilitates its entrance into host cells, while viral vector vaccines use non-replicative adenoviruses to vehiculate S-encoding DNA to the nucleus. Both membrane-bound and cytosolic pattern recognition receptors can recognize different components of the vaccines. In the case of mRNA vaccines, the lipid nanoparticle may be recognized by Toll-like receptor (TLR) 4, and mRNA by both TLR7 and melanoma differentiation-associated protein 5 (MDA5), while TLR9 may recognize DNA in adenovirus vector vaccines (reviewed in [31]).

The release of MC mediators in mastocytosis patients following vaccination has been related to the activation of TLR and non-canonical activation of FcεRI by superantigens/superallergens bound to IgE [18]. Such events have been reported in children, and rarely in adults [18,19,32]. While viral infections may activate MC through binding of viral antigens to RIG-1 or TLR and C5a to C5aR/CD88, COVID-19 infection has previously not been related to significant MC activation symptoms in mastocytosis patients [33]—a finding which has been replicated in this study. Because, in mRNA vaccines, mRNA is enclosed in a PEGylated nanolipid envelope, a potential IgE-mediated cause for anaphylaxis has been extensively studied, but conflicting findings have arisen [10,11,13,34,35,36,37]. Moreover, complement-mediated MC activation (so-called complement activation-related pseudoallergy, CARPA) induced by IgM/IgG immunocomplexes against PEG/polysorbate has also been suggested [8,13,38,39,40]. Other excipients have also been suggested as the cause of allergic reactions, namely tromethamine [12] (present in mRNA-1273 and pediatric BNT162b2 vaccines). While clonal MC activation disorders are associated with increased hypersensitivity reactions that should prompt adequate monitoring at the time of vaccination, there is no evidence that IgE-mediated sensitization to excipients in this patient population is more frequent when compared to the general population. MC from patients with systemic mastocytosis overexpress CD88 [41]; therefore, CARPA could be a cause of MC activation reactions due to COVID-19 vaccinations. Since these have been shown to be dose-dependent reactions [40], premedication could prevent reactions or at least hamper reaction severity.

Premedication protocols in mastocytosis and MCAS have not been a subject of controlled studies, are not standardized, and their preventive antimediator efficacy is unknown. Still, they are considered best practice and experts have suggested pre-medications with H1 and H2-antihistamines, leukotriene blockers and glucocorticoids before surgical procedures, general anesthesia and radiological testing using radiocontrast media [42]. While vaccination is not widely perceived as an indication for premedication [42], recommendations have been issued by the European Competence Network on Mastocytosis and the American Initiative in Mastocytosis for the use of premedication with an H1 antihistamine 30–60 min before COVID-19 vaccination [43]. However, mild MC activation symptoms or anaphylaxis have been reported in 6 out of a cumulative n of 283 (2%) patients who had premedicated with only an H1 antihistamine [23,24,25,26] and in 6 out of 57 (11% in a single study) who had not, and whose baseline antimediator therapy is unclear [25]. Except for one case in which reactions occurred following 2 doses [26], it is unclear whether these patients later tolerated further doses of the same/different vaccine platform. Of note, 3 of these patients had CM [25], which is often and mistakenly considered not to be a risk factor for anaphylaxis. Only 1 of the aforementioned studies clearly states that none of the patients (n = 73) had anaphylaxis related to prior vaccinations [26]. Here, we reported an absence of MC-related reactions in a cohort of mastocytosis patients who were vaccinated (87% premedicated), presenting with a low proportion of prior history of anaphylaxis (notably 1 had had anaphylaxis caused by a vaccine) or drug-related MC activation, who received a total of 81 doses. While it might be plausible, it is still unclear whether the absence of reactions resulted from our premedication protocol. 

Even though a small proportion of reactions might be considered normal among clonal MC disorder patients, justifying a less conservative premedication protocol (limited to an H1 antihistamine), the predicament in which we are living should warrant further consideration. Damage to public health caused by any such reactions—mild as they may be—far exceeds damage caused to patients who suffer from them, and may cause alarm over the safety of these vaccines among groups of patients with MC disorders and the general population alike. When we first used this protocol at the very beginning of the vaccination campaign [22], those vaccinated were 2 healthcare workers with mastocytosis and uncontrolled MC activation symptoms, who had daily contact with COVID-19 patients and who were scheduled for vaccination on very short notice—risk/benefit analysis clearly tended towards using a more robust premedication protocol. We chose to mimic those used for prevention of perioperative MC activation symptoms recommended for mastocytosis patients, but decided not to use glucocorticoids as they might dampen vaccine effectiveness [44,45]. Since then, our protocol and protocols similar to ours have been used by several experts [34,35,46].

Considering the limitations of our study, our findings derived from a limited cohort in which only a third of patients had a prior history of anaphylaxis. Additionally, the retrospective arm (consisting of patients who did not premedicate) was too short to be considered a proper control group and did not allow us to derive conclusions on the indications for premedication among clonal MC disorder patients. Moreover, MC activation was only assessed clinically, as we did not obtain pre- and post-vaccination serum tryptase values as other authors did [23]. There are, nonetheless, several strengths about this study, starting with this being the first study on clonal mast cell disorders that includes a high number of booster doses, patients with prior history of vaccine anaphylaxis and a preschool-aged child. Moreover, contrarily to other cohorts, ours included only 1 patient with MMAS and anaphylaxis due to Hymenoptera venom, a group of patients known to be less prone to drug-related MC activation [47]. As such, our patients could be considered at higher risk than those included in other studies. The main strength of this study is a word of hope for patients with mastocytosis (especially younger ones) and practitioners, as we have again shown that these vaccines are safe and should be used whenever indicated.

Concerning the appropriate setting for vaccination of clonal MC disorder patients, around 1 out of 3 of patients received their booster dose at ambulatory vaccination centers. While we still advocate primary vaccination in a hospital with an intensive care unit, it might be safe to perform booster dosing in an ambulatory setting.

## 5. Conclusions

Our findings amount to the current evidence that shows that COVID-19 vaccination is safe in patients with clonal mast cell disorders, underlining the fact that patients with clonal mast cell activation syndromes and mastocytosis should receive them whenever indicated (including patients with prior history of anaphylaxis following vaccination). Even though controlled studies on the need/specific indications for premedication are still lacking, our study points towards the use of a more robust premedication protocol as a means to prevent MC activation following COVID-19 vaccination. Moreover, this premedication protocol may also allow the administration of COVID-19 vaccines to patients with clonal mast cell disorders in an ambulatory setting.

## Figures and Tables

**Table 1:** Epidemiological, clinical and laboratory features of clonal mast cell disorder patients that submitted to COVID-19 vaccination.

		Prospective Arm (n = 26)	Retrospective Arm (n = 4)
**Sex (female)**		16 (62%)	3 (75%)
**Age (years)**		37 (5, 74)	50 (28, 76)
**Age at onset of mastocytosis (months, m/years, y)**		27y (2m, 74y)	43y (15y, 76y)
**Diagnosis**	MMAS	1 (4%)	0 (0%)
	CM	5 (19%)	0 (0%)
	MIS	6 (23%)	1 (25%)
	ISM	12 (46%)	1 (25%)
	BMM	1 (4%)	1 (25%)
	SSM	1 (4%)	0 (0%)
	SM-AHN	0 (0%)	1 (25%)
**Clinical manifestations of** **mastocytosis**	Skin lesions	22 (85%)	3 (75%)
	Cutaneous symptoms	23 (88%)	2 (50%)
	GI symptoms	18 (69%)	2 (50%)
	CV symptoms	13 (50%)	2 (50%)
	Anaphylaxis	9 (35%)	0 (0%)
**Triggers for mast cell activation**	Drugs	8 (31%)	0 (0%)
	Idiopathic	5 (19%)	0 (0%)
	Foods	3 (12%)	0 (0%)
	Vaccines	1 (4%)	0 (0%)
	*Hymenoptera* venom	1 (4%)	0 (0%)
**Allergic sensitization**		12 (46%)	1 (25%)
**Allergic diseases**	Asthma	4 (15%)	1 (25%)
	Allergic rhinitis	9 (35%)	1 (25%)
**Laboratory findings**	Total IgE *	27 (2, 264)	-
	sBT (ng/mL)	12 (2.4, 380)	10 (7, 11.7)
	*KIT*^D816V^ mutation **	10 (59%)	3 (100%)
**COVID-19 vaccine doses**	1	1 (4%)	0 (0%)
	2	13 (50%)	2 (50%)
	3	12 (46%)	2 (50%)
**Had COVID-19**		10 (38%)	1 (25%)

Results expressed as number of patients and percentage in parentheses (rounded to units) or as median and range in parentheses. BMM, bone marrow mastocytosis; CM, cutaneous mastocytosis; CV, cardiovascular; GI, gastrointestinal; IgE, Immunoglobulin E; ISM, indolent systemic mastocytosis; SM-AHN, systemic mastocytosis with an associated hematological neoplasm; MIS, mastocytosis in the skin; sBT, serum baseline tryptase; SSM, smoldering systemic mastocytosis. * Analyzed in 22 patients, in the prospective arm, and none in the retrospective arm; ** Analyzed in 20 patients.

## Data Availability

The data presented in this study are available from the corresponding author on request.
